# Attitude and willingness toward blood donation in Iranian students

**DOI:** 10.4103/0973-6247.53876

**Published:** 2009-07

**Authors:** Reza Afshar, Suzan Sanavi, Siamak Afshin-Majd, Sayeh Sanavi

**Affiliations:** Associate Professor of Shahed University (Faculty of Medicine), Internist, Mustafa Khomeini Hospital, Tehran; 1 Internist, Mustafa Khomeini Hospital, Italia Street, Tehran, Iran; 2 Assistant Professor of Shahed University (Faculty of Medicine), Mustafa Khomeini Hospital, Tehran; 3 Assistant to the Hejrat High School Principal, Sixteenth Education Region, Tehran

Dear Sir,

As the maintenance of safe and adequate blood supply requires recruitment, retention, and renewal of an active donor pool, we conducted two separate studies on 262 medical students of the Shahed University and 416 girls of the Hejrat High School in Iran, in the year 2009, to assess their attitude and willingness toward blood donation. The participants, 90% female and 10% male, were between the ages of 15 to 21 years. The high school students studied in four grades and three scientific courses, including:31% grade 1, 27% grade 2 (25% Natural, 27% Mathematics, 48% Humanities), 26% grade 3 (30% Natural, 34% Mathematics, 36% Humanities), and 16% grade 4 or preuniversity stage (32% Natural, 42% Mathematics, 26% Humanities), and medical students divided into three groups:(37.8%) first to second year, (45%) third to fifth year, and (17.2%) sixth to seventh year. The attitude and willingness toward blood donation were assessed by 5-point Likert scale, from 5 (strongly agree) to 1 (strongly disagree), consisting of 14 items (seven positive and seven negative toward blood donation) and the total score was calculated as the average of all items (with the results of negative items inverted).[1] The attitude of the participants was highly positive with a mean score of 4.31 ± 0.43 on the Likert scale (from 1 to 5). Blood donation willingness was declared as 87% in high school students and 89.3% in university students. Out of the participants, 16% stated that they would donate only to their relatives and the rest were ready to donate to all needy persons. There were no correlation between the age; gender; education levels; scientific courses and blood donation willingness in two student groups, but education levels and scientific courses had significant correlation with attitude toward blood donation in high school students (*P*=0.02, *P*=0.01). In comparison to other studies, Iranian students had a significantly positive attitude and willingness toward blood donation,[2,3] which indicated a proper education program in education centers, however, it is necessary to expand this education program in order to promote public awareness and willingness to donate blood .We recommend larger studies on public awareness toward blood donation.
